# Effects of urodilatin on natriuresis in cirrhosis patients with sodium retention

**DOI:** 10.1186/1471-230X-7-1

**Published:** 2007-01-26

**Authors:** Jan Carstens, Henning Grønbæk, Helle K Larsen, Erling B Pedersen, Hendrik Vilstrup

**Affiliations:** 1 Department of Medicine V, Aarhus University Hospital, Aarhus, Denmark; 2 Research Laboratory of Nephrology and Hypertension, Aarhus University Hospital and Department of Medical Research, Holstebro Hospital, Holstebro, Denmark

## Abstract

**Background:**

Sodium retention and ascites are serious clinical problems in cirrhosis. Urodilatin (URO) is a peptide with paracrine effects in decreasing sodium reabsorption in the distal nephron. Our aim was to investigate the renal potency of synthetic URO on urine sodium excretion in cirrhosis patients with sodium retention and ascites.

**Methods:**

Seven cirrhosis patients with diuretics-resistant sodium retention received a short-term (90 min) infusion of URO in a single-blind, placebo-controlled cross-over study. In the basal state after rehydration the patients had urine sodium excretion < 50 mmol/24 h.

**Results:**

URO transiently increased urine sodium excretion from 22 ± 16 μmol/min (mean ± SD) to 78 ± 41 μmol/min (P < 0.05) and there was no effect of placebo (29 ± 14 to 44 ± 32). The increase of URO's second messenger after the receptor, cGMP, was normal. URO had no effect on urine flow or on blood pressure. Most of the patients had highly elevated plasma levels of renin, angiotensin II and aldosterone and URO did not change these.

**Conclusion:**

The short-term low-dose URO infusion increased the sodium excretion of the patients. The increase was small but systematic and potentially clinically important for such patients. The small response contrasts the preserved responsiveness of the URO receptors. The markedly activated systemic pressor hormones in cirrhosis evidently antagonized the local tubular effects of URO.

## Background

Sodium and water retention occurring in decompensated cirrhosis and leading to ascites is a clinical challenge and may be difficult to treat [1-3]. Therapeutic possibilities include sodium restriction, diuretics and aldosterone antagonists, and in refractory cases repeated paracentesis, transjugular intrahepatic porto-systemic shunt (TIPS), or liver transplantation. Further pathophysiological insight and new treatment modalities are desirable. Any new drug with the potential of increasing sodium and water excretion in cirrhosis patients with refractory ascites and severe sodium retention will be a significant clinical improvement for this group of patients.

The atrial natriuretic peptide (ANP) has been investigated as such an alternative agent, but the benefits were quite modest [4-7]. Our group in previous works focused on the renal effects of synthetic urodilatin (URO), another member of the natriuretic peptide family, with dose-dependent renal effects in normal man [8,9]. Endogenous URO is the product of a different posttranslational processing of pro-ANP that takes place in the distal tubule cells. URO released into the tubular lumen stimulates natriuretic peptide receptors on the luminal surface of tubular cells and thereby inhibits distal tubular sodium reabsorption [10]. Specific guanylate cyclase-coupled receptors in the inner medullary collecting ducts are the physiological target sites for URO and cGMP is the second messenger for URO's biological actions. [11,12]. Thus, URO is an intrarenal paracrine regulator of sodium and water homeostasis. Urine urodilatin excretion is normal in patients with cirrhosis even in the presence of marked sodium retention. The coexistence of increased ANP levels and normal urodilatin excretion suggests that in cirrhosis the two natriuretic peptides are regulated independently [13].

In patients with cirrhosis and light to moderate sodium retention we earlier showed that administration of synthetic URO more than doubled natriuresis and increased diuresis due to enhanced fluid delivery from the proximal tubules, and in addition, inhibited fractional sodium reabsorption in the distal nephron, as demonstrated by lithium clearance [14].

The aim of this study was to investigate the natriuretic and diuretic effects of synthetic URO in cirrhosis patients with severe sodium retention and ascites refractory to conventional diuretic therapy.

## Methods

### Patients

The inclusion criteria were: (1) presence of cirrhosis in a liver biopsy; (2) or anamnestic, clinical and laboratory evidence of cirrhosis including ascites (verified by ultrasound), oesophagogastric varices (verified by gastroscopy), hypoalbuminemia and reduced prothrombin index; (3) a 24-h urine sodium excretion <60 mmol; and (4) written informed consent. The exclusion criteria were: (1) primary kidney disease (s-creatinine > 200 μmol/L); (2) congestive heart failure; (3) diabetes; (4) haemoglobin < 6.0 mmol/L; and (5) a history of bladder dysfunction. The withdrawal criteria during or after the study were: (1) severe adverse events during or after URO, and (2) lack of urination at the appointed time points.

Seven cirrhosis patients with ascites refractory to pharmacotherapy were consecutively recruited in our department. Their mean age was 54 ± 7 years (mean ± SD), mean body weight 69 ± 13 kg; five men and two women. Three patients had cirrhosis verified by a liver biopsy. Four patients had oesophagogastric varices. All the patients had alcoholic liver disease. The basal characteristics of the patients including their MELD score are summarized in Table 1. All patients received diuretics as a combination therapy of spironolactone and furosemide. The patients had their diuretic treatment and hydration status adjusted in the time period between enrolment and the first study infusion day. The patients had normal ECG. Plasma-creatinine was 67 ± 19 μmol/L.

Informed written consent was obtained from all patients, and the study was approved by the local Ethics Commitee of Aarhus and the Danish National Board of Health; and it was performed in accordance with the Declaration of Helsinki.

### Design

It was a randomised, single-blind, placebo-controlled study with synthetic urodilatin (Boehringer Mannheim GmbH, Mannheim, Germany). Each patient participated on two study days. Each patient received, in a randomised order, a 90-min infusion of URO (20 ng kg body weight^-1 ^min^-1^) and a 90-min infusion of placebo (PL). It thus was a cross-over design with a wash-out period of 2–3 days.

The lyophilised URO was dissolved in isotonic saline solution. Placebo was isotonic saline. Infusions were given by pump at a rate of 0.2 ml kg^-1 ^h^-1^. In a patient of eg 70 kg bodyweight, the total infusion volume was 21 mL. The URO dose was chosen from previous observations with a threshold dose of 20 ng kg body weight^-1 ^min^-1 ^and risk of side effects with higher doses [8].

### Procedures

All patients were hospitalised during the study. A 24-h urine sample was collected before enrolment. There was a run-time of four to 14 days during which diuretic treatment was adjusted and stabilized, and it was discontinued the day before infusion of both URO and placebo, but continued in between the two study days. The patients fasted from midnight and took notes of the time points of last urination before sleep and morning urination, and the urine volume including nightly urine was measured (clearance period 1). On the study day, 200 mL of water was given orally every 30 min from 7:30 am till noon. The study comprised a pre-infusion phase of 90 min (7:30 am to 9 am; clearance period 2), the infusion phase of 90 min (9 am to 10:30 am; clearance periods 3 and 4) and a post-infusion phase of 90 min (10:30 am to noon; clearance periods 5 and 6). Urine was collected in consecutive 45-min clearance periods from 9 am. The morning urine and urine collected at 9 am were used as baseline (clearance periods 1 and 2). At 9 am, infusion was initiated. The patient was in supine position, except during urination, which took place in upright position.

Urine samples from the 24-h urine and clearance periods were analysed for sodium and potassium contents. At the beginning and end of each clearance period, venous blood samples were drawn for concentrations of sodium, renin, angiotensin II (AngII), aldosterone (Aldo), and cyclic guanosine monophosphate (cGMP). Blood was secured in the supine position and before urination and followed by administration of the same volume of isotonic saline i.v. Systemic blood pressure (BP) and heart rate (HR) were measured every 20 min from 8 am till noon.

### Methods

Plasma sodium (P_Na_) and creatinine and urine sodium (U_Na_), potassium (U_K_) were determined by an autoanalyser (local Department of Clinical Biochemistry). Serum AngII, Aldo, renin and cGMP were determined by RIAs as described in detail earlier [8-10].

### Statistical analysis

We investigated seven patients and one of these was excluded from data analysis due to incomplete urine collection during the study day. Data not showing normal distribution within the same group was analysed by non-parametric Friedmans ANOVA test followed by non-parametric Willcoxon paired samples test. For unpaired comparisons between the URO and the placebo group non-parametric Kruskall-Wallis ANOVA test was used followed by the ManWhitney test. The differences between groups were based on the relative changes (Δ) from pre-infusion levels. A *P*-value of 0.05 was the limit of significance. Mean values ± SD of the absolute and relative changes (Δ) from pre-infusion levels are presented. Pearson correlation coefficient was used to measure the relationship between two variables.

## Results

### Urine sodium excretion rate (U_Na_)

**Figure 1:**
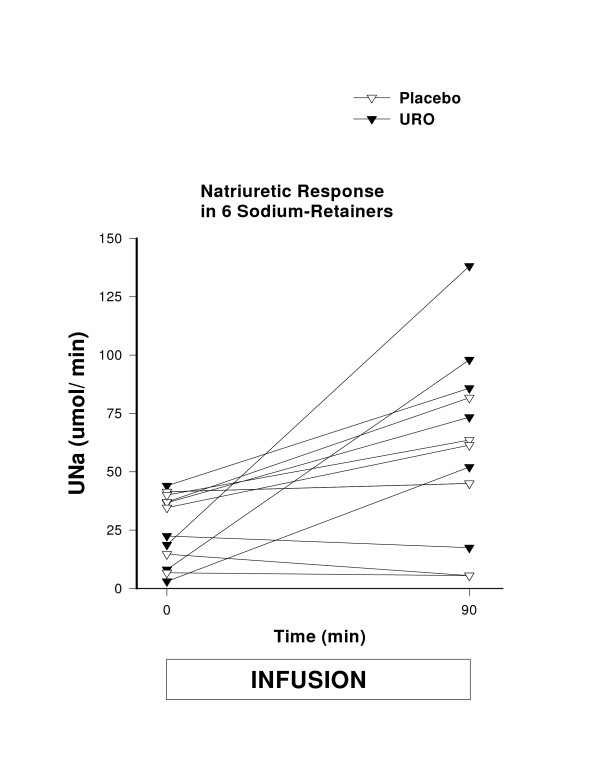
Urine sodium excretion rate (U_Na_) at baseline (pre-infusion level) and after 90 min of infusion of URO and placebo in the 6 sodium retainers. P < 0.05 URO end of infusion period compared to baseline.

**Figure 2:**
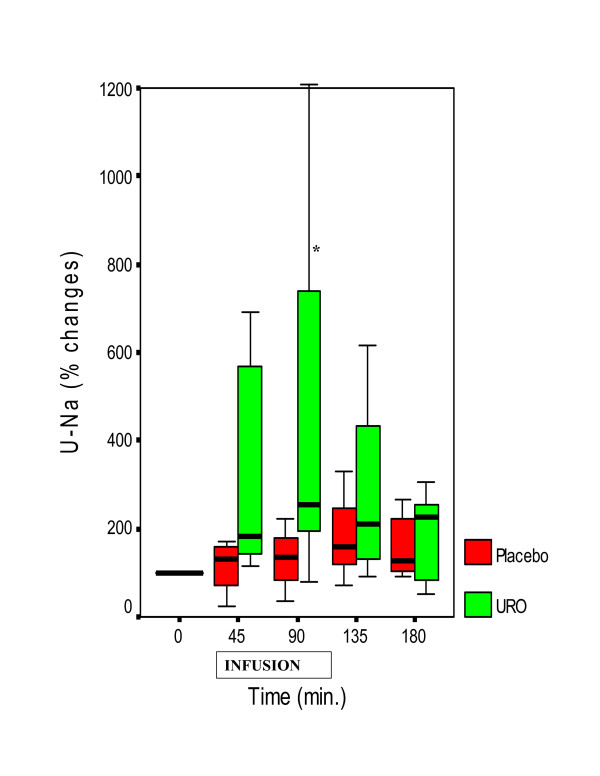
Relative changes (%) in urine sodium excretion rate (U_Na_) before (0 min), during (45 and 90 min), and after (135 and 180 min) infusion of URO and placebo in the 6 patients with severe sodium retention. Boxplots represent the interquartile range and whiskers represent highest and lowest values. * P = 0.05 URO end of infusion period compared to baseline.

**Figure 3:**
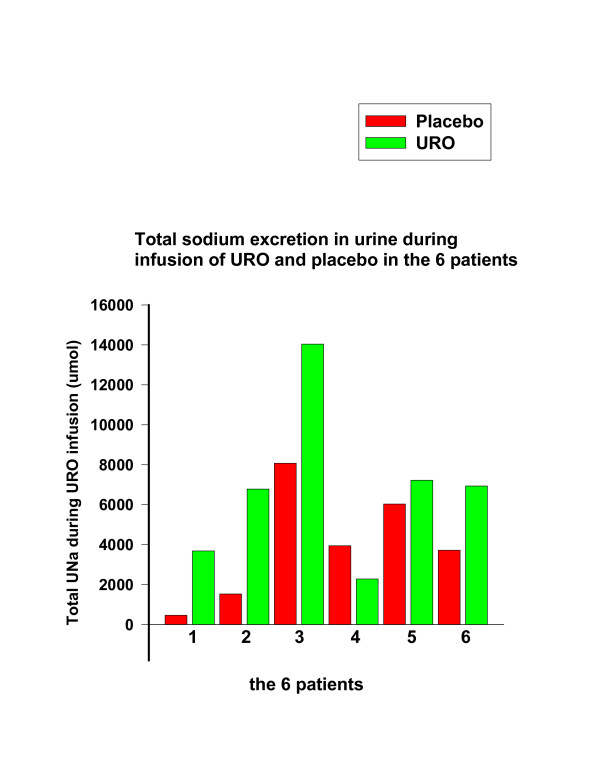
Total urine sodium excretion during URO infusion and placebo is depicted for each patient.

The average 24-hour urine sodium excretion rate (U_Na_) was 35 ± 10 mmol/day (or 24 ± 9 umol/min) before study start. U_Na _baseline values are shown in Table 1. On average, URO increased U_Na _from 22 ± 16 μmol/min (mean ± SD) to 78 ± 41 μmol/min (P < 0.05) while there was no significant effect of placebo (29 ± 14 to 44 ± 32) (fig. 1). At the end of the study, there was no difference between URO and placebo (fig. 2). The relative changes, ΔU_Na_, from baseline following URO infusion, tripled in period 3 (P = 0.10) and rose 6-fold (P = 0.05) in period 4, with very large variation. Placebo had no appreciable effect (fig 2). Total sodium excretion during URO and placebo infusions is shown in Figure 3.

### Urine flow rate (V)

**Figure 4:**
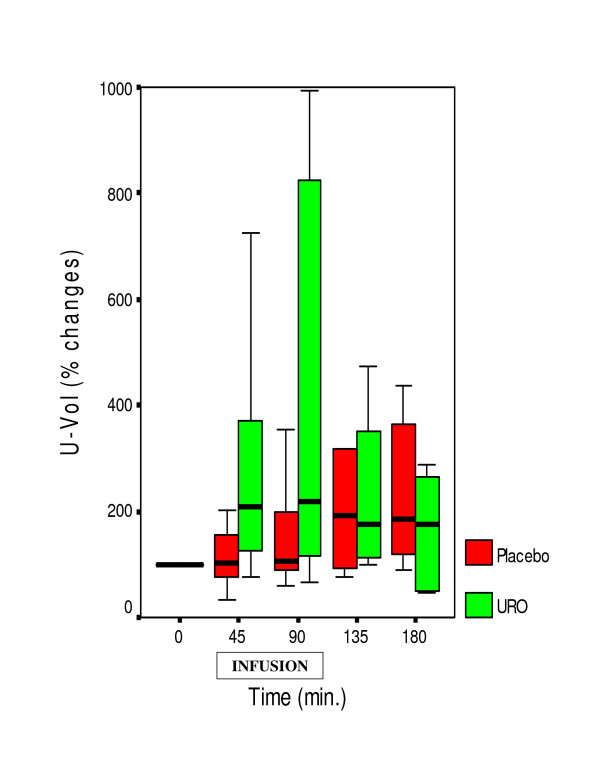
Relative changes (%) in urine flow rate (U-Vol) before (0 min), during (45 and 90 min), and after (135 and 180 min) infusion of URO and placebo in the 6 patients with severe sodium retention. Boxplots represents the interquartile range and whiskers represent highest and lowest values.

V baseline values are shown in Table 1. On average, URO increased V from 0.81 ± 0.36 ml/min to 2.95 ± 2.54 ml/min, while placebo increased V from 1.29 ± 0.58 ml/min to 2.36 ± 2.45 ml/min. The relative change in urine flow after URO was marginally larger than after placebo in period 3 (URO, 240 ± 258 %; PL, 13 ± 60%; P = 0.07), but not in period 4 (URO, 337 ± 373%; PL, 52 ± 110%; P = 0.18), see Figure 4. Following infusion stop, V tended to decrease on the URO day compared to Placebo (P = 0.1).

### Second messenger cGMP

**Figure 5:**
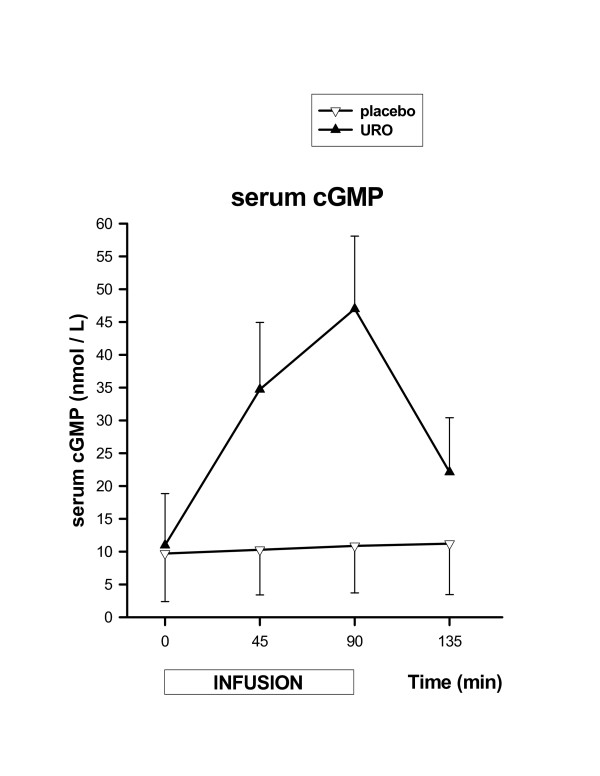
Serum cGMP before before (0 min), during (45 and 90 min), and after (135 and 180 min) infusion of URO and placebo in the 6 patients with severe sodium retention. Results are given as means ± SD. * P < 0.01 URO end of infusion period compared to baseline.

URO infusion markedly increased serum cGMP (*P *< 0.01) (fig. 5).

**Figure 6:**
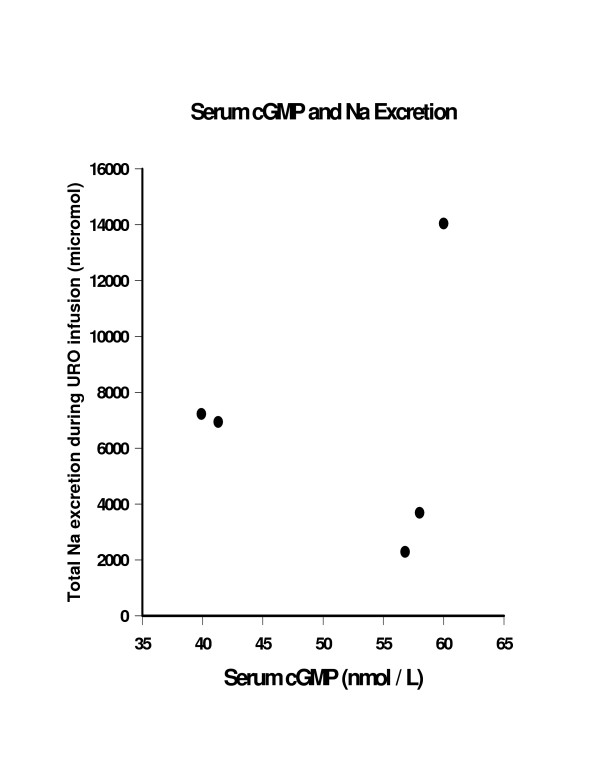
Corresponding values of serum cGMP and total sodium excretion in urine after 90 min of URO infusion. There was no significant correlation between the two variables.

The second messenger cGMP response was linked to the infusion of URO, but the serum level of cGMP achieved was independent of the level of urine sodium output, see Figure 6.

### Serum renin, angiotensin II and aldosterone

**Figure 7:**
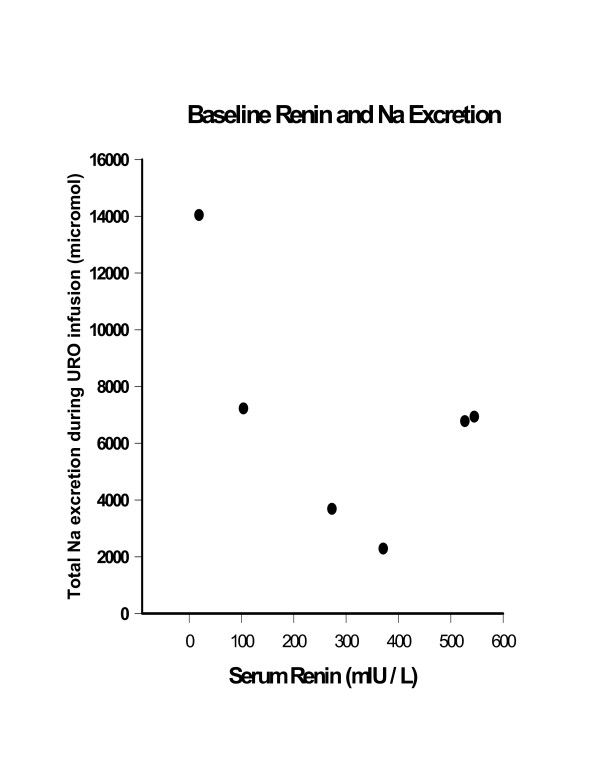
Corresponding values of baseline serum renin and total sodium excretion in urine after 90 min of URO infusion. There was no significant inverse correlation between the two variables.

**Figure 8:**
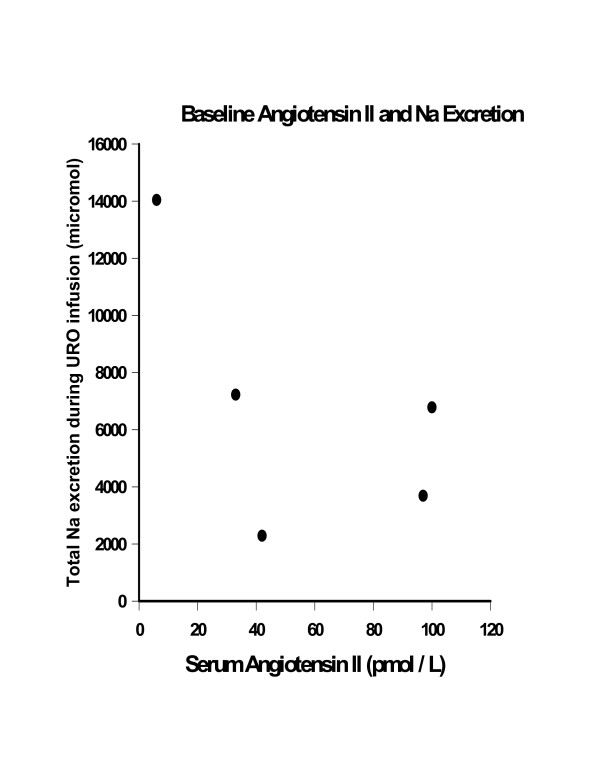
Corresponding values of baseline serum angiotensin II and total sodium excretion in urine after 90 min of URO infusion. There was no significant inverse correlation between the two variables.

**Figure 9:**
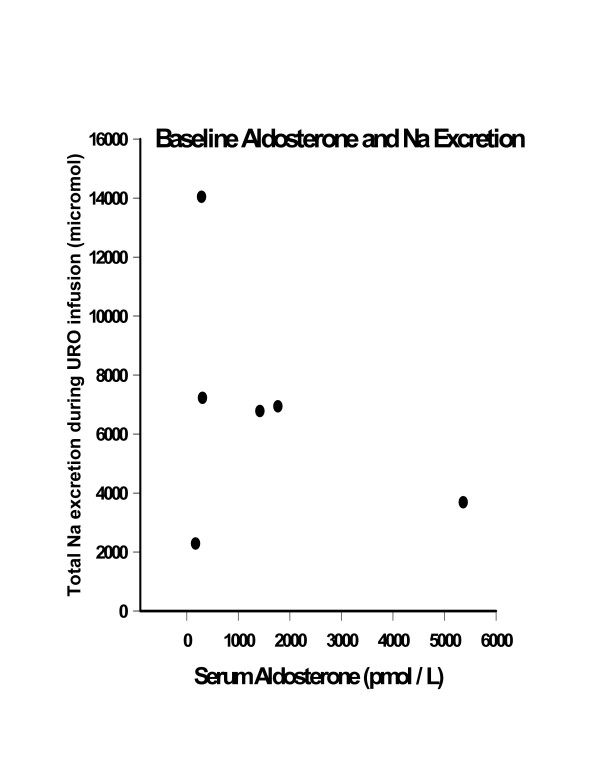
Corresponding values of baseline serum aldosterone and total sodium excretion in urine after 90 min of URO infusion. There was no significant inverse correlation between the two variable.

Pre-infusion concentrations of renin, AngII and Aldo in serum are shown in Table 1. All except one patient had highly elevated hormone levels compared to values measured in normal man [9]. Neither infusions changed any of these levels (data not shown). There was no significant inverse correlation between the baseline hormone levels and the natriuretic response to URO (fig. 7, 8, 9). However, the highest urine sodium output was measured in the only patient who had normal baseline concentration levels of all three hormones.

### Systemic Blood Pressure (BP), Heart Rate (HR) and body weight

There was no significant difference in changes in these variables between URO and placebo infusions. There was a minor decrease of body weight both days.

### Adverse effects

One patient complained of dryness in the mouth, and another vomited once in the post-infusion phase of URO. One patient had a gradual drop in BP from 95/55 to 65/50 mmHg after 85 min of URO infusion. The infusion was completed, and the patient recovered in Trendelenburg.

## Discussion

The main finding of the present study was that short-term synthetic URO infusion induced increased natriuresis in cirrhosis patients with severe sodium retention. The effect was much smaller than in our previous studies in healthy subjects and patients with less advanced cirrhosis and minor sodium retention [8,9,14]. The cGMP response to URO was normal.

In the previous study we demonstrated increased delivery of isotonic fluid from the proximal tubule and decreased fractional distal tubular sodium reabsorption during a URO-infusion, using the lithium clearance technique [14]. URO probably inhibited isotonic fluid reabsorption in the proximal tubule. Still, the mechanism of action of URO in the proximal nephron is unclear since proxinal tubule cells do not exhibit a cGMP response. The present investigation, in being a bed-side study, did not adopt radioactive tracers or the lithium clearance technique to delineate the tubular site of action of URO; we assume that the increased natriuresis was a result of both proximal and distal tubular action, as in our previous study.

The reason why the natriuretic response was weaker in the patients with refractory ascites is not clarified. Obvious differences between the patients with refractory ascites and less advanced cirrhosis are the high plasma levels of the anti-natriuretic hormones like renin, angiotensin II and aldosterone. The proximal tubular action of these anti-natriuretic hormones probably diminished distal tubular sodium delivery to the specific guanylate cyclase-coupled receptors in the inner medullary collecting ducts, the main target sites of URO. This is supported by the normal cGMP response. Likewise, the same hormones probably also antagonized the inhibitory effect of URO on isotonic fluid reabsorption in the proximal tubules. Both mechanisms would restrict normal tubular effects of URO.

The natriuretic effect of URO is mediated by cGMP, released from the URO receptor. The response of this second messenger was similar to the level measured after administration of synthetic URO in healthy subjects and in patients with less advanced cirrhosis [8,14]. This indicates intact biological responsiveness of the URO receptors in severe sodium retainers, including peptide receptor coupling and intracellular URO signalling. The normal cGMP responses both in patients with normal and decreased sodium excretion point to dissociation between the biochemical (cGMP) and the physiologic (natriuresis) response to URO. This is in line with the prevention by the pressor hormones of urine sodium reaching the URO site of action. Still, we found no relation between the level of these hormones and the natriuretic effect of URO in our patients, but this was probably due mostly to the small number of patients.

In the present study the effects of URO on urine flow were highly variable but the overall impression was that URO tended to increase diuresis (fig. 3). This is in accordance with the mode of action of the peptide where water and sodium handling are coupled. In the same way as regarding natriuresis, the aquaretic effect of URO was much larger and more consistent in cirrhosis patients with less sodium retention [14].

The demonstrated renal pharmacodynamic effects of URO were short-lived (fig. 2) because of the short half-life (about 5 min) of URO. Therefore, there is also no reason to suspect a carry-over effect between study days.

We used a dose of 20 ng URO kg^-1 ^min^-1 ^since higher doses may lower systemic blood pressure [8,9]. Accordingly, there was no appreciable effect on blood pressure. Adverse effects were minor except in one patient with a moderate fall of systolic blood pressure at the end of the URO infusion. A similar low frequency of adverse effects was observed in our previous study [14].

The patients continued on their adjusted medication and their usual fluid and food intake until the day before study start in order to study them in a clinically and physiologically stable phase, and also to minimize the discomforts of taking part in a study. The patients were not placed on a sodium controlling diet, but the identical baseline (pre-infusion) levels of urine sodium excretion on both study days suggest that the patients were in adequately stable sodium equilibrium. We stopped their diuretics first on the day before the infusion of either URO or placebo so that spironolactone and its metabolites may still have antagonized the tubular action of aldosterone and increased the delivery of sodium to the distal nephron and the site of action of URO and contributed to the increase in natriuresis. The cross-over design with an interval of two-three days between infusions likely, however, ensured identical conditions on both study days.

The mild increase of sodium excretion rate in the placebo group is well-known in clearance studies with water-loaded subjects. It is of no importance in healthy subjects responding vividly to URO, but it calls for special care in the interpretation of the natriuretic effect of URO in the presented sodium retainers.

The clinical implications of URO are not clarified. Stimulation of the sympathetic nervous system and activation of the renin-angiotensin-aldosterone system play a pivotal role in the resistance to conventional diuretics in advanced cirrhosis. As mentioned above, similar forces are most likely the cause of the relative resistance to URO. Maneuvres that increase distal tubular sodium delivery may restore the renal responsiveness to URO in advanced cirrhosis. If so, URO is not potent alone, but may be so in combination with loop of Henle diuretics and distal tubular diuretics.

We are aware of the limitations of the study as a result of the small number of patients. Larger trials are needed to confirm the results before it is possible to discuss the clinical use of URO in cirrhosis patients. The clinical value of URO has been investigated in other diseases and mostly with disappointing outcomes. URO was found to have no beneficial effect in acute renal failure or in prevention of renal impairment following liver transplantation [15,16]. In a recent study on decompensated chronic heart failure a 24-hour infusion of URO substantially decreased cardiac filling pressure because of the vasodilatory effects, but no improved renal function was reported [17].

The URO infusion transiently shifted our patients from a stage with threatening hepatorenal syndrome to a stage with controllable salt balance. Although the demonstrated natriuretic effect was physiologically small, it may nonetheless be clinically important by lifting the patients over the threshold of irreversible decompensation. Alternatively, the results can be taken to show that the mechanisms that favour sodium excretion seem to be operative if the sodium retaining systems can be de-activated.

## Conclusion

These patients with decompensated cirrhosis and severe sodium retention responded systematically but modestly to a short-term low-dose URO infusion compared to the large natriuretic response observed in previous studies with healthy subjects and patients with less advanced cirrhosis. The only small natriuretic and diuretic response contrasted the preserved biologic responsiveness of the URO receptors in the inner medullary collecting ducts. This was probably due to the activation of the systemic mechanisms behind sodium and water retention in cirrhosis, evidently antagonizing the tubular effects of URO.

## Competing interests

The author(s) declare that they have no competing interests.

## Authors' contributions

JC have contributed to acquisition of data, analysis and interpretation of data and have been involved in drafting the manuscript.

HG have contributed to acquisition of data, made substantial contributions to analysis and interpretation of data as well as being involved in drafting the manuscript.

HKL have made substantial contribution to acquisition of data.

EBP have made contribution to interpretation of data, critical revision of the manuscript and technical/material support.

HV conceived of the study, participated in its design and coordination, helped to critical revise manuscript and gave administrative and technical/material support.

All authors read and approved the final manuscript.

## Pre-publication history

The pre-publication history for this paper can be accessed here:



## Figures and Tables

**Table 1:** The basal characteristics of the 6 patients included in the statistical analysis.

	No. 1	No. 2	No. 3	No. 4	No. 5	No. 6
[unit/reference interval]	Male	Male	Female	Male	Male	Female
Total Plasma Bilirubin [< 22 micromol/L]	33	549	35	33	17	38
Plasma Albumin [551–725 micromol/L]	366	445	330	506	272	294
PP [0.80–1.20 arbitrary units]	0.55	0.20	0.30	0.66	0.42	0.39
Ascites	yes	yes	yes	yes	yes	yes
Encephalopathy	no	no	no	no	no	no
MELD score [5–15 points]	6	27	9	6	6	9
Plasma Sodium [136–146 mmol/L]	132	134	140	135	125	132
Plasma Creatinine [55–120 micromol/L]	105	50	62	85	52	58
Urine Sodium/24 hours [mmol/24 h]	10	35	27	48	35	31
UNa (baseline) [μmol/min]	URO 3.1 Pl. 6.7	URO 28.1 Pl. 14.8	URO 36.7 Pl. 37.0	URO 22.5 Pl. 41.4	URO 44.0 Pl. 40.0	URO 18.7 Pl. 34,7
V (baseline) [mL/min]	URO 0.44 Pl. 1.04	URO 0.35 Pl. 0.44	URO 1.20 Pl. 1.77	URO 0.74 Pl. 0.90	URO 1.18 Pl. 1.65	URO 0.93 Pl. 1.93
Serum Renin [10–70 mIU/L]	273	527	19	371	104	545
Serum Angiotensin II [10–30 pmol/L]	97	100	6	42	33	
Serum Aldosterone [40–500 pmol/L]	5363	1419	287	171	306	1768
Systemic Blood Pressure [mmHg]	95/60	112/65	100/60	112/65	105/80	100/65
Furosemide [mg/24 hours]	80	40	40	120	80	80
Spironolactone [mg/24 hours]	200	200	200	200	200	200

URO = urodilatin. Pl. = placebo. UNa = urine sodium excretion rate. V = urine flow rate. PP = prothrombin time. MELD = Model of End-stage Liver Disease.
